# Thermal equation of state of Molybdenum determined from in situ synchrotron X-ray diffraction with laser-heated diamond anvil cells

**DOI:** 10.1038/srep19923

**Published:** 2016-02-17

**Authors:** Xiaoli Huang, Fangfei Li, Qiang Zhou, Yue Meng, Konstantin D. Litasov, Xin Wang, Bingbing Liu, Tian Cui

**Affiliations:** 1 State Key Lab of Superhard Materials, College of physics, Jilin University Changchun 130012, P.R. China; 2 High-Pressure Collaborative Access Team, Argonne National Laboratory, Carnegie Institution of Washington, Argonne, Illinois 60439, USA; 3 Department of Geology and Geophysics, Novosibirsk State University, Novosibirsk 630090, Russia; 4 V. S. Sobolev Institute of Geology and Mineralogy, SB RAS, Novosibirsk 630090, Russia

## Abstract

Here we report that the equation of state (EOS) of Mo is obtained by an integrated technique of laser-heated DAC and synchrotron X-ray diffraction. The cold compression and thermal expansion of Mo have been measured up to 80 GPa at 300 K, and 92 GPa at 3470 K, respectively. The *P*-*V*-*T* data have been treated with both thermodynamic and Mie–Grüneisen-Debye methods for the thermal EOS inversion. The results are self-consistent and in agreement with the static multi-anvil compression data of Litasov *et al.* (J. Appl. Phys. 113, 093507 (2013)) and the theoretical data of Zeng *et al.* (J. Phys. Chem. B 114, 298 (2010)). These high pressure and high temperature (*HPHT*) data with high precision firstly complement and close the gap between the resistive heating and the shock compression experiment.

High-pressure studies on materials have attracted a great enthusiasm, which allows tuning the atomic, electronic structure and also produces novel materials. The pressure value needs to be obtained from the diffraction line shifts in a standard material which is mixed with the sample and whose pressure–volume -temperature (*P*-*V*-*T*) equation of state (EOS) is well known^1^. One of the most important issues is how to accurately estimate the pressure values especially at ultrahigh pressure and temperature conditions^2^. Accurate thermal EOS for solid materials can directly provide valuable information of their phase diagrams and dynamical responses under extreme conditions^3^,^4^. So far, the accurate thermal EOS for some transition metals, such as Ti, Ta, W, and Fe, have been performed by theoretical or experimental methods^5^,^6^. As a body-centered-cubic (bcc) 4*d* transition metal, Mo has been the subject of extensive theoretical and experimental investigations focusing on its melting curve and the solid-solid phase transition under high pressure^7^,^8^. However, there is little data of *P*-*V*-*T* EOS for Mo especially determined from the experimental diffraction measurements. Furthermore, theoretical studies are expected to be further confirmed by X-ray diffraction (XRD) study. Here, we have obtained the EOS of Mo up to 100 GPa and 3000 K by an integrated technique of laser-heated DAC and synchrotron XRD.

There are several experimental factors, which may cause the disagreements between the EOS: pressure scales, EOS formalism, pressure-transmitting medium (PTM), and especially experimental methods. Previous studies focused on shock wave experiments or theoretical methods to obtain the EOS of Mo, but till now few static experiments have been done^9^,^10^,^11^,^12^,^13^. The two recent static experiments reported by Zhao *et al.*^14^ and Litasov *et al.*^15^ have measured the *P*-*V*-*T* EOS for Mo with *in situ* synchrotron XRD or neutron-diffraction techniques. The data of Zhao *et al.* were obtained by a DIA-type cubic anvil press up to 10 GPa and 1475 K, with NaCl as the pressure scale and PTM^14^. Litasov *et al.* extended the *P*-*T* conditions up to 31 GPa and 1673 K, which were conducted by using a Kawai-type multi-anvil apparatuses^15^. Nevertheless, the *P*-*T* ranges of these investigations are lower than those generated with the laser-heaed diamond anvil cell (DAC) techniques. The *in situ* laser-heated DAC has been a unique static technique for reaching ultrahigh *P-T* conditions (*P* *>* 100 GPa, *T* *>* 1500 K), leading to numerous important discoveries and novel phenomena^16^,^17^. Recently, the laser-heated DAC in conjunction with synchrotron radiation sources has undergone rapid development and become a powerful tool for the EOS measurements^18^,^19^. And the issue of an axial temperature gradient in the sample layer has been resolved by introducing the double-sided laser heating technique.

In this work, we have performed *in situ* synchrotron XRD measurements integrated with the double-side laser-heated DAC techniques to obtain the *P*-*V*-*T* EOS of Mo to higher precision and at higher *P*-*T* conditions. Neon (Ne) has been used as the PTM for generating better hydrostatic pressure condition. The least controversial MgO pressure scale was used as the internal standards under high pressure and temperature. High temperature data have been treated with both thermodynamic and Mie–Grüneisen-Debye methods for the thermal EOS inversion. The present technique with higher precision complements the data gap between the multi-anvil apparatuses and the shock compression experiments.

## Results and Discussion

**Figure 1:**
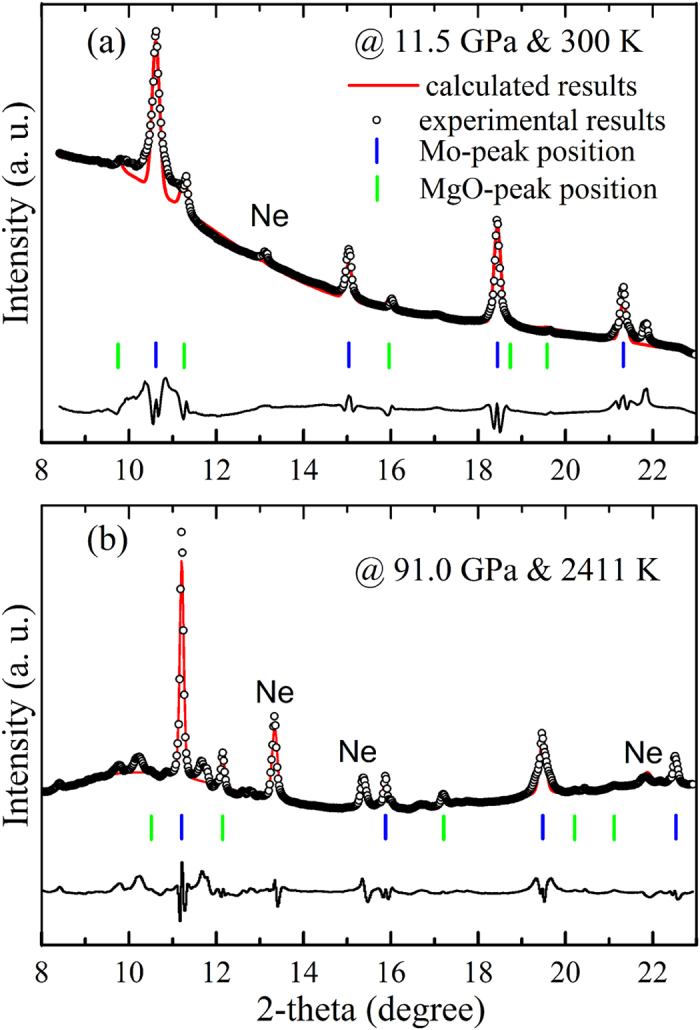
Representative XRD pattern at high *P*-*T*. (**a**) At 11.5 GPa & 300 K and (**b**) at 91.0 GPa & 2411 K.

Representative XRD patterns from one heating cycle are shown in Fig. 1 and the peaks of PTM Ne are marked. Figure 1(a) is a typical XRD pattern before heating. Since the unidentified weak peaks in the diffraction patterns existed after gas loading, it is considered that these unknown peaks maybe from impurities acquired during gas loading. These weak peaks in the diffraction patterns cannot be identified for now. However, since the XRD of target sample Mo and MgO can be well distinguished from the whole XRD pattern, and both phases are stable up to the highest pressure and temperature. For the purpose of this work, the unidentified peaks can be ignored. In the seven experimental runs of this study, no new XRD peaks appeared, indicating that no chemical reaction occurs or products are produced during all of the heating cycles. For both Mo and MgO, there are at least four peaks for each phase to calculate the lattice constants and volume under high pressure and temperature, as shown in Fig. 1(b).

As pointed out by Fei *et al.*^20^, MgO is considered as the most useful pressure scale in practice, because its EOS is least controversial. Recently, Sokolova *et al.*^21^ revised the thermal EOS of MgO, which is generally consistent with other pressure scales such as ruby, diamond and metals. In this work, for all of the compression runs, MgO scale proposed by Sokolova *et al.*^21^ was used as the internal standards under high pressure and temperature.

**Figure 2:**
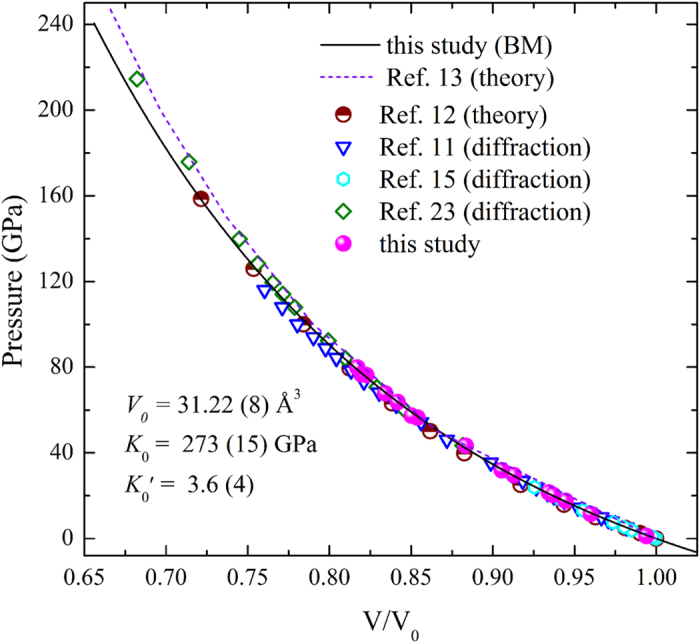
Summary of 300 K isotherm of Mo measured in this study, compared with previous experimental and theoretical results. The solid black curve represents the BM EOS fit to the experimental data of this study.

The synchrotron XRD data of Mo was collected up to 80 GPa at room temperature. At these pressures, the sample Mo remained in bcc phase with space group *Im*-3*m*. The data for ambient temperature EOS of Mo are plotted in Fig. 2. The choice of the EOS at 300 K is critical for determining the parameters of the thermal EOS model from the measured thermal pressure. Therefore, to provide useful physical parameters, the *P*-*V* data points have been fitted by the third-order Birch-Murnaghan (BM) EOS^20^, which yields ambient volume *V*_0_ = 31.22 ± 0.08 Å^3^, isothermal bulk modulus *K*_0_ = 273 ± 15 GPa, and its pressure derivative *K*_0_′ = 3.6 ± 0.4. Although the shock Hugoniot is typically considered as the most accurate “primary EOS standard”, the data accuracy of the shock compression is not be completely trusted as pointed out by Chijioke *et al.*^22^. Figure 2 shows the reported static experimental and theoretical results comparing with our experimental results. The data collected from the theoretical calculations by Wang *et al.*^12^ perfectly fall on our fitted curves. Recently, Zeng *et al.*^13^ have performed a systematic study of the thermal EOS using the theoretical calculation. Their calculated volumes at the same pressure are higher than those in this study especially above 100 GPa. For the *P*-*V* data at 300 K determined from the XRD patterns, the data of Litasov *et al.*^15^ are in agreement with our results, while the data of Dorfman *et al.*^23^ and Dewaele *et al.*^11^ are slightly deviated from our fitted BM EOS in this study. Litasov *et al.* measured the volume of Mo up to 31 GPa and obtained the pressure values from MgO by Sokolova *et al.*^21^, using the same pressure scale with this study. Although Dorfman *et al.* have created a good hydrostatic pressure condition with helium as a PTM, and used the MgO scale proposed by Tange *et al.*^24^, the volume obtained by Dorfman *et al.* was about 1.6% higher than those in present study at 200 GPa. At the highest pressure of 116 GPa measured by Dewaele *et al.*, the obtained volume was about 0.8% lower than this study. The pressure for Mo by Dewaele *et al.* was estimated from the pressure calibration of the ruby ball. The minor deviation among Dorfman *et al.*, Dewaele *et al.* and this study is maybe attributed to the different pressure scale. It is known that different pressure scales are able to generate large uncertainty in calculating the thermal pressure, and in some cases, the calculated pressures based on different standards could differ as much as 4 GPa^20^. Therefore, the small pressure differences between Dewaele *et al.* and this study are reasonable.

Tsuchiya *et al.*^25^ have reported that the electronic thermal pressure is nearly independent of volume and have presented the *P*_el_ (*T*) values as a function of *T*, for instance, *P*_el_ (*T*) = 0.04, 0.21 and 1.60 GPa at 300, 1000 and 3000 K, respectively. So the electronic contributions to its free energy can be neglected. In this case, the thermal EOS of a solid normally has the following form as: 26,27


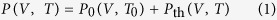


in which the electron thermal contributions are considered to be negligible compared to the ion thermal component in the range of this study. The subscript 0 refers to ambient conditions. The left side of this equation represents the total pressure *P* at volume *V* and temperature *T*. The *P*_0_ (*V*, *T*_0_) corresponds to the static pressure along the ambient temperature isotherm, and the *P*_th_ (*V*, *T*) is the isothermal pressure at high temperature. For most solids, the *P*_0_ (*V*, *T*_0_) can be well determined by the BM EOS. For the *P*_th_ (*V*, *T*), there are usually two approaches (via thermodynamic or Mie-Grüneisen-Debye formalism) used for calculating the thermal pressure *P*_th_ (*V*, *T*) with the static compression experimental data. Firstly, in the thermodynamic approach, the *P*_th_ (*V*, *T*) beyond the 300 K isothermal is conveniently evaluated by integration at constant volume presented as:^27^


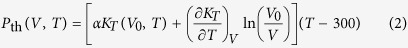


Therefore, in the thermodynamic approach, the pressure determined in the Eq. (1) becomes as follows:


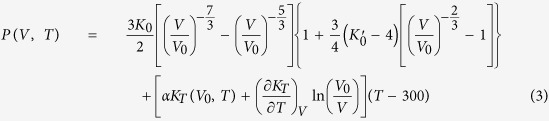


Secondly, for the Mie-Grüneisen-Debye (MGD) approach, the thermal pressure can be obtained as follows:^27^


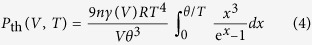


where *n* is the number of atoms per formula unit, *γ* is the Grüneisen parameter, *R* is the gas constant, and *θ* is the Debye temperature. The Grüneisen parameter *γ* is assumed to be independent of temperature and its volume dependence is


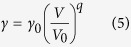


where Grüneisen parameter *γ* is a function of volume only with *q* = *d*ln*γ*/*d*ln*V*. The parameter *q* was previously taken to be 1 implying *γ*/*V* = const. This commonly accepted formulation has been changed recently. In this study, the parameter q is fitted to be 0.6.

The Debye temperature *θ* with the following form is related to the volume change.


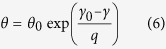


In the Mie-Grüneisen-Debye approach, the thermal EOS has the following form as:


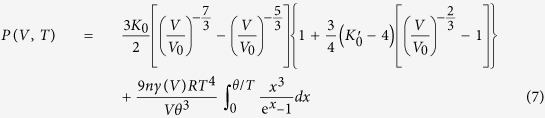


**Figure 3:**
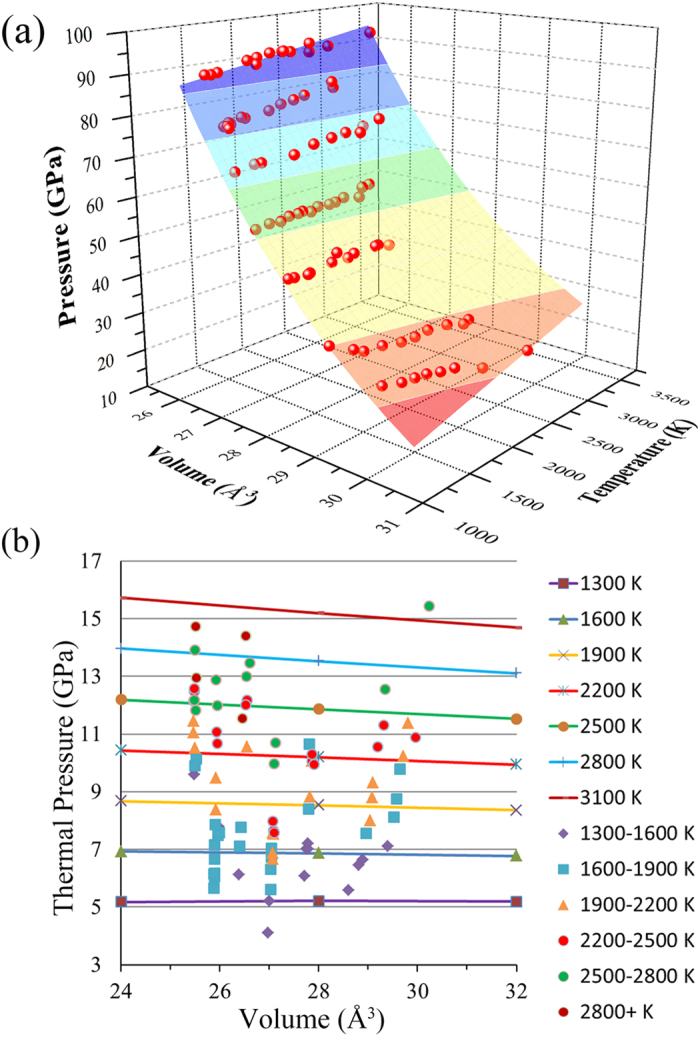
Summary of *P*-*V*-*T* data measured in this study. (**a**) The fitted surface represents the thermodynamic EOS fit to the experimental data of this study. (**b**) The measured *P*_*th*_ – *V* - *T* data with solid symbols are fitted by MGD EOS. The Solid lines are fitted isothermal compression curves at 1300, 1600, 1900, 2200, 2500, 2800, and 3100 K, respectively.

**Figure 4:**
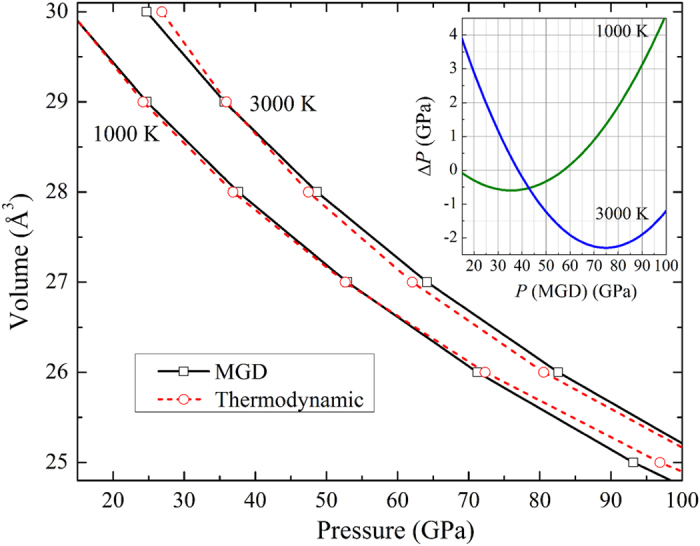
Comparison of the EOSs of Mo obtained with thermodynamic and MGD approach for this study. Inset figure shows the pressure differences between the thermodynamic EOS and the MGD EOS at selected temperature of 1000 K, and 3000 K, respectively.

Both thermodynamic and MGD approaches were used for the *P*-*V*-*T* EOS inversion in this study. For all the experimental runs, the data points are directly measured at each temperature (listed in Table 1), and are fitted through these two approaches, as shown in Fig. 3. The thermodynamic EOS fitting yields *K*_*0*_ = 231 ± 6 GPa, *K*_0_′ = 5.7 ± 0.3, *αK*_*T*_ (*V*_*0*_, *T*) = 0.007 ± 0.0004 GPa/*K* and (*∂K*_*T*_/*∂T*)_*V*_ = –0.016 ± 0.003 GPa/*K* with fixed *V*_0_ = 31.14 Å^3^ for all the data of Mo measured under high pressure and high temperature. The MGD fitting is performed with fixed Debye temperature *θ*_0_ = 470 K^28^,^29^, because the fitting of experimental *P*-*V*-*T* data yields unrealistically high Debye temperature. The final parameters for the best fit to MGD approach in this study are listed in Table 2 along with previous results. The fitted parameters for both thermodynamic and MGD approaches are obtained, so the isothermal *P*-*V* data at any desired temperature can be calculated either from Eq. (3) or Eq. (7) by using the fitted parameters. It is important to compare the isotherms obtained from thermodynamic and MGD approaches (Fig. 4). As the theory indicated, the results from these two approaches should be consistent with each other. Our results show that they are in good agreement with each other below 100 GPa. The maximum pressure deviation between these two EOSs is about 3.0 GPa below 100 GPa. The argument has been made that the MGD method is preferable because it better represents the thermoelastic properties and provides a more secure basis for interpolating or extrapolating the results beyond the studied *P*-*T* ranges^27^.

**Figure 5:**
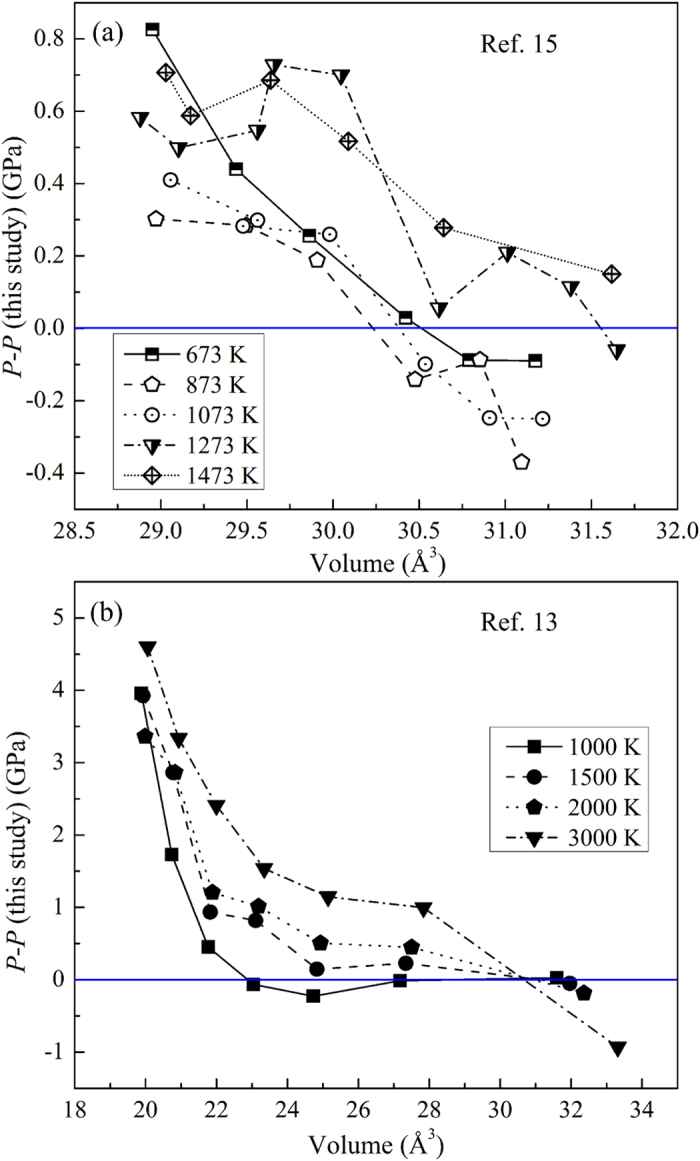
Pressure differences between the present EOS of Mo with previous results. Pressure differences between the present EOS of Mo and (**a**) the EOS by Litasov *et al.*^15^, and (**b**) the EOS by Zeng *et al.*^13^ at selected isotherm, respectively.

In Fig. 5, the pressure differences are shown between the present EOS of Mo and the EOS by Litasov *et al.*^15^, and the EOS by Zeng *et al.*^13^ at selected isotherms, respectively. The EOS of Mo from Zeng *et al.* is calculated using density-functional theory. Litasov *et al.* have used ultra-hard 26 mm WC anvils to generate high pressure and high temperature conditions monitored by a thermocouple located at nearly the same position as where the X-rays pass through the sample. In Fig. 5(a), we found that the pressure deviation between Litasov *et al.* and this study is lower than 1.0 GPa, and the data of Litasov *et al.* are in remarkable agreement with ours. Owing to the limitation of the apparatus used by Litasov *et al.*, they only measured the volume of Mo to a relative lower *P*-*T* range within 30 GPa and 1500 K. Figure 5(b) shows the comparison between the EOS obtained from the theoretical calculations from Zeng *et al.* and this study. Below 100 GPa, these two EOSs give a small pressure deviation of 1.0 GPa. Above 100 GPa, Zeng *et al.* gives higher pressure than this study and the maximum pressure difference reaches about 5 GPa for the 3000 K isotherm, notably the pressure deviation becomes larger with increasing temperature. It is difficult to account the reason for this discrepancy, however, it is important to note that theoretical calculations may provide significant error at highest temperature due to uncertainty in the accounting for the electronic contributions to thermal pressures and may need further improvements. This study is the first *in situ* laser-heated DAC experiment for the EOS of Mo, which has extended the *P*-*T* conditions up to 92 GPa and 3470 K. The present technique with higher precision helps to close the data gap between the resistive heating experiments in large volume apparatuses and the shock compression experiments. Besides, the crystal structure of bcc Mo is confirmed up to 94 GPa and 3470 K without any evidence for the predicted transition to a close-packed face-centered cubic (fcc) phase in the *P*-*T* range.

## Conclusion

In summary, Mo is studied by an integrated technique of DAC, laser-heating and synchrotron XRD, providing experimental insight into its behavior at high pressure and temperature. We have measured the cold compression of Mo with the Ne pressure media up to 80 GPa, and its thermal expansion up to 92 GPa and 3470 K. The third-order BM EOS of Mo at room temperature are fitted with ambient volume *V*_0_ = 31.22 (8) Å^3^, isothermal bulk modulus *K*_0_ = 273 (15) GPa, and its pressure derivative *K*_0_′ = 3.6 (4). High temperature data have been treated with both thermodynamic and Mie–Grüneisen-Debye methods for the thermal EOS inversion. The present EOS of Mo can be used as a reliable pressure scale for static experiments up to 100 GPa and 3000 K.

## Experimental Methods

Seven static compression experiments were conducted at the 16ID-B beamline of Advanced Photon Source (APS) in Argonne National Laboratory (ANL) using double-sided laser-heated DACs. Firstly, the sample preparation is so important that it directly influences the temperature stability. The starting sample consists of the mixture of Mo and MgO powder. Beveled anvils with both 300 *μm* and 200 *μm* culets are used to generate lower pressure and higher pressure, respectively. Before loading the powder sample into the sample chamber, we compressed the powder into flakes by using the DAC. We have adopted the sample loading with three rubies as a divider between the sample and each diamond anvil. The sample is insulated from the anvils to guarantee the heating homogeneity. Then Neon (Ne) was loaded into the sample chamber using COMPRES/GSECARS gas-loading equipment^30^, which served as a thermal insulator and PTM. Under this condition, the sample was suspended in the Ne surroundings to guarantee good hydrostatic condition and thermal insulation. According to the melting curve of Ne^31^, in some of our experimental runs, the melting temperature of Ne is really well below some of the high temperature Mo data, so Ne is partially liquid. This will have positive effect in our experiment, since liquid Ne will provide a better hydrostatic pressure than solid Ne.

Secondly, the temperature control in LHDAC is a challenging question, and we have tried several times to obtain the stable temperature during the LHDAC. During each compression run, the sample was compressed with certain pressure point and then was heated with Nd: YLF laser to high temperature for several minutes. With a fiber size of 100 *μm* in diameter and a 50 *μm* entrance slit, thermal radiation from a 5 × 5 *μm*^2^ hot spot is collected for temperature measurement. Furthermore, the heating spot on the sample, as well as the coupling between the heating spot and the temperature measurement, can be monitored from both sides using CCD cameras and adjusted as needed. The heating temperature is uniform across the sample with the difference lower than 20 K. The temperatures reported for each diffraction pattern are from the peak intensities at the center of the hotspot and the average temperature of both sides is used with an average uncertainty of ∼50 K, although this double-sided laser-heated DAC system is optimized for laser heating and temperature measurement. The same situation is also observed in other LHDAC experiments. For example, Lazicki *et al.*^32^ has performed a work on the phase diagram and the equation of state of beryllium using LHDAC, and he has mentioned that the temperature was determined from spectral radiometry measurements with an average uncertainty of ∼100 K. Besides, the temperature stability over the duration of the XRD measurement is also important parameters influencing the precision of the *P*-*V*-*T* data. During the duration of the XRD measurement, the temperature is monitored all the time and is confirmed to be stable with fluctuation of ∼10 K, which also can be seen from the recent report^33^.

The angle-dispersive XRD patterns of the sample were collected on an imaging plate with an exposure time of 1 min for every heated point under high pressure. The monochromatic incident X-ray beam with a wavelength of 0.4066 Å was collimated to 6 × 7 *μ*m^2^ while the laser heating spot was about 48 *μ*m in diameter. Two-dimensional XRD images were integrated as a function of 2*θ* angle in order to provide a conventional diffraction pattern using the Fit2D program^34^. Corresponding temperature measurements from each side of the sample and XRD patterns were obtained at 1 min intervals throughout the course of each temperature cycle, for a total of about 20 diffraction patterns and temperature profiles over 20–30 min.

## Additional Information

**How to cite this article**: Huang, X. *et al.* Thermal equation of state of Molybdenum determined from in situ synchrotron X-ray diffraction with laser-heated diamond anvil cells. *Sci. Rep.* **6**, 19923; doi: 10.1038/srep19923 (2016).

## Figures and Tables

**Table 1:** The measured lattice parameters and volumes of MgO and Mo at different temperatures and pressures.

*T*(K)	MgO			Mo	
	*a*(Å)	*V*(Å^3^)	*P*(GPa)	*a*(Å)	*V*(Å^3^)
Mo + MgO + Ne_1					
1486(18) ()()(0	4.0921(4)	68.52(2)	23.7	3.0864(1)	29.40(2)
1633(23)	4.0993(12)	68.89(8)	23.4	3.0905(5)	29.52(1)
1748(32)	4.1033(20)	69.09(12)	23.5	3.0922(15)	29.57(4)
1842(31)	4.1050(28)	69.17(14)	23.8	3.0948(7)	29.64(2)
1954(34)	4.1121(15)	69.54(8)	23.4	3.0976(5)	29.72(2)
2065(39)	4.1150(9)	69.68(4)	23.6	3.1006(9)	29.81(3)
2311(45)	4.1386(82)	70.88(42)	21.6	3.1060(12)	29.96(3)
2693(12)	4.1421(33)	71.06(17)	23.4	3.1147(33)	30.22(9)
Mo + MgO + Ne_2					
1355(31)	4.0439(7)	66.13(4)	31.5	3.0584(5)	28.61(1)
1502(33)	4.0571(14)	66.78(7)	29.9	3.0655(1)	28.81(4)
1570(32)	4.0642(7)	67.13(4)	29.1	3.0683(2)	28.89(1)
1730(39)	4.0688(6)	67.36(3)	29.2	3.0709(2)	28.96(1)
1901(47)	4.0774(9)	67.79(4)	28.7	3.0736(3)	29.04(1)
2028(48)	4.0809(4)	67.96(2)	28.9	3.0756(5)	29.09(1)
2172(20)	4.0840(7)	68.12(3)	29.3	3.0759(2)	29.10(1)
2344(18)	4.0895(1)	68.39(1)	29.4	3.0793(4)	29.20(1)
2474(6)	4.0978(17)	68.81(8)	28.8	3.0835(8)	29.32(2)
2517(26)	4.0940(10)	68.62(5)	29.7	3.0845(2)	29.35(1)
Mo + MgO + Ne_3					
1389(11)	3.9841(9)	63.24(4)	44.3	3.0262(2)	27.71(5)
1426(6)	3.9844(1)	63.25(4)	44.5	3.0729(2)	27.76(4)
1559(35)	3.9894(9)	63.49(5)	44.2	3.0286(3)	27.78(8)
1579(49)	3.9887(11)	63.46(5)	44.4	3.0287(1)	27.78(1)
1800(26)	3.9911(12)	63.57(6)	45.3	3.0292(2)	27.80(1)
1850(66)	3.9829(12)	63.20(6)	47.4	3.0295(3)	27.81(1)
1961(1)	3.9951(14)	63.77(7)	45.3	3.0305(4)	27.83(1)
2014(30)	3.9931(13)	63.67(6)	46.1	3.0316(3)	27.86(1)
2256(40)	4.0000(16)	64.00(8)	46.1	3.0316(4)	27.86(1)
2281(24)	4.0005(15)	64.02(7)	46.2	3.0319(6)	27.87(2)
2394(26)	4.0070(17)	64.34(8)	45.4	3.0328(3)	27.90(1)
Mo + MgO + Ne_4					
1414(3)	3.9435(20)	61.33(9)	54.0	2.9962(3)	26.90(1)
1538(74)	3.9458(22)	61.43(10)	54.6	2.9999(1)	27.00
1641(62)	3.9488(23)	61.57(11)	54.5	3.0009(2)	27.03(1)
1729(78)	3.9480(21)	61.54(10)	55.2	3.0013(1)	27.03(1)
1818(87)	3.9497(22)	61.62(10)	55.3	3.0019(1)	27.05
1864(83)	3.9499(23)	61.62(11)	55.6	3.0020(1)	27.05(1)
1939(22)	3.9537(24)	61.80(11)	55.1	3.0026(2)	27.07(1)
1944(1)	3.9546(24)	61.84(11)	54.9	3.0025(2)	27.07(1)
2017(1)	3.9528(23)	61.76(11)	55.8	3.0026(1)	27.07
2142(4)	3.9566(24)	61.94(11)	55.6	3.0031(1)	27.08
2210(23)	3.9577(26)	61.99(12)	55.7	3.0031(2)	27.08(1)
2312(19)	3.9584(26)	62.02(13)	56.2	3.0027(1)	27.07
2477(31)	3.9662(29)	62.39(14)	55.3	3.0039(3)	27.1(1)
2512(68)	3.9570(5)	61.96(2)	57.7	3.0037(5)	27.1(1)
2585(6)	3.9573(12)	61.97(5)	58.1	3.0045(5)	27.12(1)
Mo + MgO + Ne_5					
1478(63)	3.9018(3)	59.40(1)	66.3	2.9772(5)	26.39(1)
1674(82)	3.9034(4)	59.47(2)	67.1	2.9777(5)	26.40(1)
1731(84)	3.9042(3)	59.51(1)	67.2	2.9786(7)	26.4(2)
2025(10)	3.9083(3)	59.70(2)	67.8	2.9834(5)	26.55(1)
2242(79)	3.9078(4)	59.67(2)	69.4	2.9833(8)	26.55(2)
2460(6)	3.9110(2)	59.82(1)	69.8	2.9822(1)	26.52(3)
2614(5)	3.9123(3)	59.88(1)	70.4	2.9827(4)	26.54(1)
2759(10)	3.9174(8)	60.12(4)	69.8	2.9852(14)	26.60(4)
3031(18)	3.9155(4)	60.03(2)	72.0	2.9825(3)	26.53(1)
2870(34)	3.9170(6)	60.10(3)	70.6	2.9795(1)	26.45
Mo + MgO + Ne_6					
1592(9)	3.8731(8)	58.10(4)	75.9	2.9618(4)	25.98(1)
1603	3.8729(1)	58.09(1)	76.0	2.9609(3)	25.96(1)
1767(20)	3.8717(5)	58.04(2)	77.4	2.9592(4)	25.91(1)
1668(30)	3.8733(18)	58.11(5)	76.3	2.9580(4)	25.88(1)
1811(45)	3.8739(13)	58.14(6)	76.9	2.9585(3)	25.90(1)
2053(27)	3.8762(14)	58.24(6)	77.7	2.9596(2)	25.92(1)
2184(28)	3.8756(22)	58.21(10)	78.8	2.9595(5)	25.92(1)
2313(70)	3.8761(13)	58.23(6)	79.4	2.9605(8)	25.95(2)
2439(86)	3.8766(12)	58.26(5)	80.0	2.9604(7)	25.94(2)
2771(38)	3.8763(13)	58.24(6)	82.2	2.9595(8)	25.92(2)
Mo + MgO + Ne_7					
1593(18)	3.8372(2)	56.50(1)	88.0	2.9399(5)	25.41(1)
1643	3.8383(8)	56.55(3)	87.9	2.9441(16)	25.52(4)
1697(35)	3.8379(6)	56.53(3)	88.4	2.9434(5)	25.41(1)
1717(81) (18)	3.8389(3)	56.58(1)	88.1	2.9434(16)	25.50(4)
1811(32)	3.8396(6)	56.60(2)	88.5	2.9405(5)	25.42(1)
2037(22)	3.8396(12)	56.61(5)	89.9	2.9424(11)	25.47(3)
2141(30)	3.8403(5)	56.64(2)	90.3	2.9422(9)	25.47(2)
2250(49)	3.8603(17)	57.52(7)	84.2	2.9415(3)	25.45(1)
2283(6)	3.8409(7)	56.66(3)	91.0	2.9422(8)	25.47(2)
2411(8)	3.8433(7)	56.77(3)	90.9	2.9430(12)	25.49(3)
2429(80)	3.8433(19)	56.77(8)	91.0	2.9428(11)	25.49(3)
2721(9)	3.8456(7)	56.87(3)	92.1	2.9434(9)	25.50(2)
2119(1)	3.8444(8)	56.82(3)	88.7	2.9434(7)	25.50(2)
2507(1)	3.8461(6)	56.89(3)	90.6	2.9430(3)	25.50(1)
2711(86)	3.8521(13)	57.16(6)	89.8	2.9440(4)	25.51(1)
2932(21)	3.8534(15)	57.22(7)	90.7	2.9440(4)	25.52(1)
3470(10)	3.8573(10)	57.39(4)	92.7	2.9437(2)	25.51(1)

The pressures are calculated from the EOS of Sokolova *et al.*^21^ for MgO with *V*_*0*_ = 74.71 Å^3^. The average lattice parameters for each *P*-*T* point are obtained by arithmetic average of multiple diffraction lines.

**Table 2:** Thermoelastic parameters for Mo obtained using MGD EOS.

Parameter	Litasov *et al.*^15^	Sokolova *et al.*^21^	Zeng *et al.*^13^	This study
*V*_0_(Å^3^)	31.14^f^	31.14^f^	31.14^f^	31.14^f^
*K*_0_(GPa)	260^f^	249	245 (1)	255 (1)
*K*_0_′	4.21^f^	4.47	4.66 (1)	4.25 (2)
*γ*_0_	2.03	1.98	1.97 (2)	2.01 (2)
*q*	0.24	1.99	0.82 (3)	0.6 (2)
*θ*_0_ (K)	455–470^f^	470^f^	470^f^	470^f^

^f^Fixed according to optimized parameters and reference data.
